# Comparative Proteomic Analysis of the Diatom *Phaeodactylum tricornutum* Reveals New Insights Into Intra- and Extra-Cellular Protein Contents of Its Oval, Fusiform, and Triradiate Morphotypes

**DOI:** 10.3389/fpls.2022.673113

**Published:** 2022-03-21

**Authors:** Coralie Chuberre, Philippe Chan, Marie-Laure Walet-Balieu, François Thiébert, Carole Burel, Julie Hardouin, Bruno Gügi, Muriel Bardor

**Affiliations:** ^1^UNIROUEN, Laboratoire Glyco-MEV EA4358, Normandie Université, Rouen, France; ^2^UNIROUEN, PISSARO Proteomic Facility, Institute for Research and Innovation in Biomedicine, Normandie Université, Mont-Saint-Aignan, France; ^3^Normandie University, UNIROUEN, INSERM US 51, CNRS UAR 2026, HeRacLeS-PISSARO, Rouen, France; ^4^UNIROUEN, Institute for Research and Innovation in Biomedicine, Normandie Université, Rouen, France; ^5^Polymers, Biopolymers, Surface Laboratory, UMR 6270 CNRS, University of Rouen, Mont-Saint-Aignan, France; ^6^Institut Universitaire de France, Paris, France

**Keywords:** diatom, *Phaeodactylum tricornutum*, microalgae, secretome, proteome, morphotype

## Abstract

*Phaeodactylum tricornutum* is an atypical diatom since it can display three main morphotypes: fusiform, triradiate, and oval. Such pleomorphism is possible thanks to an original metabolism, which is tightly regulated in order to acclimate to environmental conditions. Currently, studies dedicated to the comparison of each morphotype issued from one specific strain are scarce and little information is available regarding the physiological significance of this morphogenesis. In this study, we performed a comparative proteomic analysis of the three morphotypes from *P. tricornutum*. Cultures highly enriched in one dominant morphotype (fusiform, triradiate, or oval) of *P. tricornutum* Pt3 strain were used. Pairwise comparisons highlighted biological processes, which are up- and down-regulated in the oval (e.g., purine and cellular amino acid metabolism) and triradiate morphotypes (e.g., oxido-reduction and glycolytic processes) compared to the fusiform one used as a reference. Intersection analysis allowed us to identify the specific features of the oval morphotype. Results from this study confirmed previous transcriptomic RNA sequencing observation showing that the oval cells present a distinct metabolism with specific protein enrichment compared to fusiform and triradiate cells. Finally, the analysis of the secretome of each morphotype was also performed.

## Introduction

*Phaeodactylum tricornutum* is a pleomorphic raphid pennate diatom that exists naturally under three main morphotypes: fusiform, oval, and triradiate (Lewin et al., 1958; Borowitzka and Volcani, 1978; Johansen, 1991). The oval morphotype, which is preferentially benthic, possesses a raphe and organized silicified frustule unlike the fusiform and triradiate cells, which are more planktonic species (Tesson et al., 2009; Vartanian et al., 2009). This pleomorphism, together with physiological and metabolic flexibilities, have been hypothesized to be responsible for the great adaptability of *P. tricornutum* to various environments (Rushforth et al., 1988; Gutenbrunner et al., 1994; De Martino et al., 2007; Butler et al., 2020; Falciatore et al., 2020; Song et al., 2020). Previous studies attempted to understand *P. tricornutum* polymorphism and demonstrated that such morphogenesis is not dependent on *P. tricornutum* genotype but can be induced by environmental factors (De Martino et al., 2007). Several works investigated recently the functional diversity of the ten ecotypes isolated from *P. tricornutum* that might also be a factor influencing the polymorphism (Rastogi et al., 2018, 2020; Song et al., 2020; Scarsini et al., 2021). The fusiform morphotype is the most frequent one in natural waters and *in vitro* cultures (Volcani, 1981; De Martino et al., 2007). In contrast, the triradiate are favored in an unstressed planktonic environment and preferentially developed with alkaline conditions (Johansen, 1991; De Martino et al., 2007; Bartual et al., 2008) whereas the oval cell growth rate increase in unfavorable and stress conditions (Borowitzka and Volcani, 1978; Johansen, 1991; De Martino et al., 2011). As morphotypes seem to be influenced by environmental conditions and as significant differences in the proteome regulation of *P. tricornutum* were reported in response to environmental conditions such as iron starvation (Allen et al., 2008), dark stress (Bai et al., 2016), or nitrogen deprivation (Yang et al., 2014; Longworth et al., 2016; Remmers et al., 2018), therefore, it is tempting to the hypothesis that proteomes might be different in the three morphotypes of *P. tricornutum*.

Previous comparative transcriptomic analyses of expressed sequence tags (EST) have suggested that the oval morphotype could be the most resistant form to stresses as this morphotype presents an ability to survive with limited nutrient availability and up-regulated genes encoding proteins involved in hyposalinity and cold stress responses (De Martino et al., 2007). Recently, *P. tricornutum* Pt3 strain was adapted to generate cultures enriched in one dominant morphotype: fusiform, triradiate, or oval. These cultures were used to run high-throughput RNA sequencing. The whole mRNA transcriptome of each morphotype was determined and pairwise comparisons highlighted biological processes and molecular functions, which are up- and down-regulated specifically (Ovide et al., 2018). In this previous study, less than 1% of genes were differentially expressed between the fusiform and the triradiate morphotypes whereas 22 and 29% were differentially expressed when comparing the oval morphotype *versus* the fusiform one and the oval morphotype *versus* the triradiate one, respectively (Ovide et al., 2018). Moreover, the metabolism of the oval cells was suggested to be specific compared to the other morphotypes (Ovide et al., 2018). Recently, Song et al. (2020) also reported that the oval cells synthesized a higher amount of proteins and pigments compared to the fusiform cells while the fusiform cell cultures accumulated lipids and carbohydrates. In addition, Galas and collaborators have shown that the oval Pt3 cells of *P. tricornutum* are secreted proteins more rapidly than the fusiform and triradiate Pt3 cells (Galas et al., 2021).

However, in this context, it remains unclear how the morphotype of *P. tricornutum* can impact the proteome. As previously suggested, it is essential to understand the interplay between the major players, e.g., genes (genomics), RNA (transcriptomics), proteins (proteomics), and metabolites in a cell type in order to decipher completely its cell biology (Heydarizadeh et al., 2014) and in the case of *P. tricornutum* its morphogenesis. Therefore, such a proteomic analysis will provide useful information to understand the pleiomorphism of the diatom *P. tricornutum* and could highlight morphotype-specific proteome signatures.

In this work, we performed a comparative proteomic analysis on the three morphotypes issued from the same *P. tricornutum* Pt3 strain. To reach this goal, cultures highly enriched in one dominant morphotype (fusiform, triradiate, or oval) of *P. tricornutum* Pt3 strain were used to prepare total protein extracts from cells representing the overall intracellular proteins. Then, label-free and iTRAQ^®^ quantitative proteomics were applied to compare the proteome profile of cells issued from the three morphotypes. In addition, we also analyzed the proteins secreted in the culture medium of each morphotype by the label-free approach to characterize *P. tricornutum* secretomes of each morphotype.

## Materials and Methods

### Experimental Design and Setup

Diatom cells of the Pt3 strain (CCAP 1052/1B; CCMP 2558), initially derived as a subclonal culture of Pt2 in Plymouth (De Martino et al., 2007), were grown at 19°C under a 16 h/8 h light/night cycle. Fusiform and triradiate cells were cultivated in sterilized 100% natural seawater (SW) (33.3 g/L, Instant Ocean, Aquarium System, Sarrebourg, France) and oval cells were cultivated in 10% SW (3.3 g/L). SW was then complemented with a nutrient medium (Conway 1 mL/L) and a metasilicate sodium solution (80 mg/L) (Ovide et al., 2018). Typical confocal and transmission electron microscopy (TEM) images of each morphotype can be found in Ovide et al. (2018) and Galas et al. (2021). In this work, the cultures were non-axenic. Particular attention has been paid to minimizing the presence of cellular debris in the culture medium. To do so, cells were pre-cultured (250 mL) twice in flasks under orbital agitation (150 rpm) on an IKA KS 260 Basic shaker (Sigma, St Quentin Fallavier, France) for 4 days in order to reach an exponential growth phase. Then, the cells were spun down and washed with fresh medium (SW) in order to remove cellular debris. Finally, the washed cell pellets were used to run a 1 L bioreactor using the culture conditions as previously described. Five biological replicates were performed and enriched cultures in one specific and dominant morphotype, as described in Table 1, reflecting the homogeneity of the samples.

**TABLE 1:** Relative percentage of each specific morphotype in *P. tricornutum* enriched culture.

Dominant morphotype	Morphotype enrichment* of *P. tricornutum* cultures
Fusiform	90 ± 1.2%
Triradiate	77 ± 0.5%
Oval	98 ± 0.4%

*Enrichments are expressed in specific morphotype cells number per 100 of total cells. Means were calculated over five biological replicates ± SE.

Cell type proportions were estimated using an optical microscope associated with a manual cell count using a Nageotte cell (*n* = 5).

In order to establish the different proteomes from the three main morphotypes of *P. tricornutum*, intracellular proteins and proteins secreted within the culture media, later called secretome in this paper, were independently extracted and analyzed.

### Intracellular Proteome Extraction

Total proteins were extracted from Pt3 cultures enriched in one specific and dominant morphotype. Microalgae cells from the different cultures were recovered by centrifugation at 4,500 × *g* for 5 min.

Cell pellets were washed twice with 10% SW in order to decrease salt concentration. For each culture, a pellet of approximately 1.10^8^ cells was harvested and immediately re-suspended in 500 μL of D2R2 protein extraction buffer {7 M urea, 2 M thiourea, 2 mM tributyl phosphate (TBP), 0.5% 3-(4-heptyl) phenyl-3-hydroxypropyldimethylammoniopropanesulfonate (C7BzO), and 2% 3-[(3-cholamidopropyl) dimethylammonio]-1-propanesulfonate (Chaps)}. The cell suspension was stored at –80°C until further use. For protein extraction, the cell suspension was transferred into a 2 mL tube of lysing matrix E (MP Biomedicals, Fisher Scientific, Illkirch, France), and cells were lysed by 4 runs of 30 s at 6.5 m.s^–1^ with the FastPrep^®^- 24 high-speed benchtop homogenizer (MP Biomedicals^®^). Cell lysates were centrifuged at 10,000 × *g* for 10 min in order to remove cellular debris. The supernatants containing proteins were collected. The remaining pellet was re-suspended in 500 μL of D2R2 protein buffer and extracted once more. Finally, the supernatants were pooled in a unique fraction called IP for intracellular proteins.

### Secreted Proteins From the Culture Medium

For the analysis of the secretomes (proteins secreted in the culture medium), a volume of 28 mL of each culture was centrifuged at 100,000 × *g* for 1 h at 4°C allowing removal of residual cell debris and eventual extracellular vesicles that could be present in the culture media. The supernatant was concentrated in the Pierce™ protein Concentrator 3K MWCO (Fisher Scientific, Illkirch, France) by centrifugation at 4,000 × *g* for 30 min and washed twice with deionized water. Therefore, all peptides below 3 kDa were lost during this step and only higher proteins have been analyzed. Collected supernatants containing secreted proteins, later called SP, were lyophilized, and then resuspended in deionized water prior to proteomic analysis.

### Polyacrylamide Gel Electrophoresis and Protein Staining

A sodium dodecyl sulfate-polyacrylamide gel electrophoresis (SDS-PAGE) was performed to monitor the quality of the protein extraction. Samples were loaded on a precast Bolt™ 4–12% Bis-Tris Plus Gel (Invitrogen, Fisher Scientific, Illkirch, France). after denaturation in 5X Laemmli buffer for 5 min at 100°C. Electrophoresis was performed at room temperature for approximately 45 min using a constant voltage of 200 V in a 1X solution of NuPAGE MOPS SDS running buffer (Invitrogen, Fisher Scientific, Illkirch, France) until the dye front reached the end of the gel. Proteins were stained using an home-made Coomassie blue R-250 solution (30% ethanol, 10% acetic acid, and 0.02% Coomassie R-250).

### Proteomic Sampling and Preparation

Collected supernatants called IP (640 μL) and SP (100 μl) were mixed with 5X Laemmli Buffer (0.312 M Tris (sans HCl) pH 6.8, 50% v/v glycerol, 10% w/v SDS, 5% v/v B-mercaptoethanol, and 0.25% bromophenol blue) and heated at 100°C for 10 min. Samples (600 μL for IP and 80 μL for SP) were loaded on a 7% bis-acrylamide gel. Electrophoresis was performed at 10°C for approximately 3 h using a constant amperage of 20 mA in a Tris-Glycine buffer (1.4% w/v glycine, 0.3% w/v Tris base, and 0.1% w/v SDS). Gels were washed twice in deionized water and incubated for 30 s in the Coomassie blue R-250 solution under shaking. The protein band just above the migration front was cut off and stored in a mix of 30% ethanol and 10% acetic acid until further analysis. Excised protein bands either from IP or SP have been reduced in 5 mM Tris (2-carboxyethyl) phosphine hydrochloride (TCEP, Sigma, Saint-Quentin Fallavier, France) in 100 mM TEAB at 56°C for 1 h as previously described in Young et al. (2015). Then, reduced cysteine residues were blocked using 55 mM iodoacetamide (Sigma, Saint Quentin Fallavier, France) at room temperature for 20 min before performing in-gel trypsin digestion according to the manufacturer’s instructions (Promega, Charbonnières-Les-Bains, France). The peptides were extracted once with formic acid 1%, twice with 100% acetonitrile/5% formic acid (v/v) and combined fractions dried using a vacuum concentrator (Young et al., 2015). Finally, peptide samples were resuspended in 100 μl of deionized water. Moreover, 10 μl of IP samples were used for label-free proteomic analysis, and 30 μl of IP were subjected to iTRAQ^®^ labeling according to the manufacturer’s recommendations (AB Sciex SAS, Villebon Sur Yvette, France). The three different morphotype samples: fusiform, oval, and triradiate were respectively labeled, overnight at room temperature, by iTRAQ^®^ 117/115/116 in a 3Plex experimental design. Samples from each biological replicate were finally pooled and dried-down before lnanoliquid chromatography coupled with electrospray ionization tandem mass spectrometry (nanoLC-MS/MS) analysis.

### NanoLC-ESI-MS/MS Analysis

Before running the nanoLC-ESI-MS/MS analysis, all peptide samples were resuspended in 5% (v/v) acetonitrile and 0.1% (v/v) formic acid: IP label-free (15 μL), IP iTRAQ^®^ labeling (100 μL) and SP (12 μL). Two microliters of each sample was then analyzed on the Q-Exactive Plus (Thermo Scientific, Les Ulis, France) equipped with a nanoESI source. Peptides were loaded onto an enrichment column [C18 Pepmap100 (5 mm × 300 μm i.d., 5 μm, 100 Å), Thermo Scientific, Les Ulis, France] and separated on an EASY-spray column [(50 cm × 0.075 mm i.d., 3 μm, 100 Å), Thermo Scientific, Les Ulis, France] with a flow rate of 300 nL.min^–1^. The mobile phase was composed of H_2_O/0.1% formic acid (buffer A) and acetonitrile/H_2_O/0.1% formic acid (80/20) (buffer B). The elution gradient duration was 120 min following different steps: 0–84 min, 2–35% B; 84–94 min, 35–90% B; 94–105 min, 90% B; 106–120 min, 2% B. The temperature of the column was set at 40°C. The mass spectrometer acquisition parameters were: 100 ms maximum injection time, 1.6 kV capillary voltage, 275°C capillary temperature, full scan MS *m*/*z* @ 400–1,800 with a resolution of 70,000 in MS and 17,500 in MS/MS. The 10 most intense ions (Top 10) were selected and then fragmented with nitrogen as a collision gas (normalized collision energy set to 27 and 38 eV for iTRAQ^®^). All spectra obtained were exported in “raw” format that was used for data analysis.

### Proteomic Data Analysis

Label-free and iTRAQ^®^ analyses were performed to identify and quantify the differential expression levels of intracellular proteins. Label-free approach analysis was performed to identify the secretomes. For intracellular proteins, datasets were normalized, counted prior to identification using Mascot^1^ and Progenesis software as compared to the *P. tricornutum* Uniprot database^2^.

For the quantification and the identification of the differentially regulated intracellular proteins of each morphotype, Progenesis liquid chromatography-mass spectrometry (LC-MS) software (Nonlinear Dynamics, version 4.1^3^) was used for label-free peptide approach as previously described (Fréret et al., 2013). Automatic alignment was set to perform two by two comparisons between samples in order to align the LC-MS runs to account for retention time drifts. A minimum of 80% alignment score was required for further analysis. After alignment, statistical analysis was performed with one-way ANOVA calculations. To highlight differentially expressed peptides between groups (triradiate or oval morphotype against fusiform used as a reference), an ANOVA *p*-value ≤ 0.05 was required. The peak list containing the differential expressed peptides were then used for identification using Mascot (Matrix Science, version 2.5, Boston, MA, United States) with the following parameters: enzyme specificity, trypsin; one missed cleavage permitted; variable modifications, carbamidomethylation (C); oxidation (M), pyro glu from E and Q; monoisotopic precursor mass tolerance: 5 ppm; product mass tolerance: 0.02 Da against the *P. tricornutum* Uniprot reference proteome which was cleaned by removing pseudogenes. A positive match was considered when it was ranked among the first positions and presented a score with a significant threshold of *p* < 0.05 and with a false discovery rate (FRD) below 1. Mascot search results were imported back into Progenesis for differential expression and only proteins with an ANOVA *p*-value ≤ 0.05 and identified by ≥2 peptides were retained. The quality of the data was checked by principal component analysis (PCA) (Supplementary Figure 1).

For protein identification in SP and iTRAQ^®^ labeling IP samples, peak lists were extracted (merge MS^n^ scans with the same precursor at ±30 s retention time window and ±50 ppm mass tolerance) and compared with specific databases by using the PEAKS studio 7.5 proteomics workbench (Bioinformatics Solutions Inc., Waterloo, Canada, build 20150615). The searches were performed with the following specific parameters: enzyme specificity, trypsin; three missed cleavages permitted; fixed modification, carbamidomethylation (C); variable modifications, oxidation (M), pyro-glu from E and Q; monoisotopic; mass tolerance for precursor ions, 5 ppm; mass tolerance for fragment ions, 0.02 Da; MS scan mode FT-ICR/Orbitrap; MS/MS scan mode, Linear ion Trap; Fragmentation mode, high energy CID; databases, *P. tricornutum* databases (UniProt Phaeodactylum + AND + tricornutum + AND + %22%28strain + CCAP + 1055%2F1%29%22 and Ensembl release 41 ASM15095v2.pep.03092018 [see test footnote 2]). Database search results were used for quantitative analysis with PEAKS Q and iTRAQ-4plex as selected methods. Only significant hits with a false discovery rate (FDR ≤ 1) for peptide and protein cut off (–logP ≥ 20 and unique peptides ≥ 2) and fold change > 1.2 (iTRAQ^®^ hit map) were considered. On this dataset, less than 0.5% of peptides presented a miss-cleavage greater than 1.

### Functional Annotation

**FIGURE 1:**
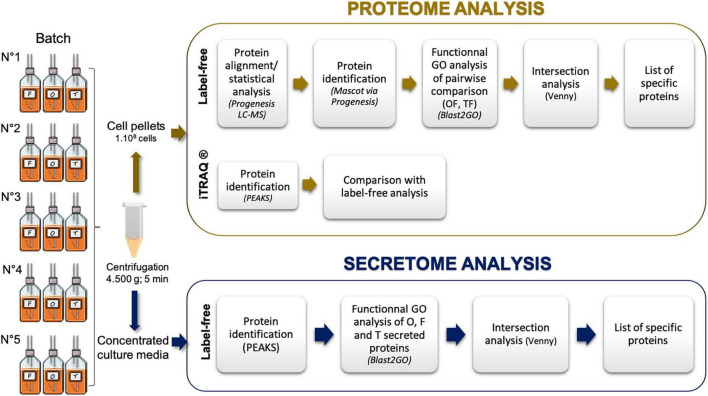
Experimental design used in the current study to identify the intracellular proteomes and secreted proteins of the three main morphotypes of *Phaeodactylum tricornutum*. F, fusiform morphotype; O, oval morphotype; T, triradiate morphotype. Five biological replicates have been performed and summed for the data analyses.

The steps implemented to perform functional annotation are summarized in Figure 1. They lead to a functional Gene Ontology (GO) analysis: GO annotation and GO enrichment were performed using Blast2GO software version 3.3.536^4^). Protein sequences in FASTA were obtained with dataset *P. tricornutum* ASM15095v2 (Ensembl protist genes 47^5^). Homology searches have been launched against NCBI nr Database (QBlast). Then, default parameters were used to map, annotate, and run InterPro scan^6^. Pie charts were done using the Multi-Level option in Blast2GO considering the biological process (Ovide et al., 2018). Intersection analysis was performed using the Venny version 2.1 (Oliveros, 2007).

Pairwise comparison of the morphotype’s intracellular proteomes was handled by considering that the fusiform morphotype was the reference as it is described to represent the most common morphotype (De Martino et al., 2007, 2011; Ovide et al., 2018).

### Overrepresentation Analysis

An overrepresentation test was performed using the PANTHER classification system (PANTHER version 15^7^; Mi et al., 2013). UniProtKB: UniProt accession sublists of selected proteins either from the fusiform, oval or triradiate morphotypes were loaded on PANTHER and compared to IDs from Reference Proteome Genome (*P. tricornutum* PHATC) using GO-slim PANTHER biological process. Fisher test and FDR correction were applied. The outcomes summarized in a Microsoft^®^ Excel^®^ spreadsheet, Microsoft, United States were used to draw the related final figures.

### Prediction of Signal Peptide and *N*-Glycosylation Sites on Secreted Proteins

The prediction of a potential signal peptide on the secreted proteins has been analyzed by the SignalP version 5^8^ (Almagro Armenteros et al., 2019). The probability was considered correct when the score was greater than 0.5. The online tool NetNGlyc^9^ has been used to predict potential *N*-glycosylation sites of the secreted proteins. This dataset was then compared to the ones reported in the studies of Rastogi et al. (2018) and Dorrell et al. (2021).

## Results

*Phaeodactylum tricornutum* cell cultures enriched in one dominant morphotype: Fusiform, Oval, and Triradiate were used in this work. Such cultures have been characterized recently using TEM and confocal microscopies (Ovide et al., 2018; Galas et al., 2021). Especially, the latest study demonstrated that the three morphotypes share similarities in terms of organelle localization (nucleus, mitochondria, F-actin cortex, intracellular network, etc.). In contrast, compared to fusiform and triradiate cells, oval cells spontaneously release proteins more rapidly, underlying a more rapid protein secretion in the oval morphotype (Galas et al., 2021). In the present study, we analyzed through proteomic the intracellular proteins and secreted proteins from the different morphotypes issues from the same Pt3 strain (Figure 1).

### Differences in Intracellular Protein Productions Exist Between the Three Pt3 *Phaeodactylum tricornutum* Morphotypes

**FIGURE 2:**
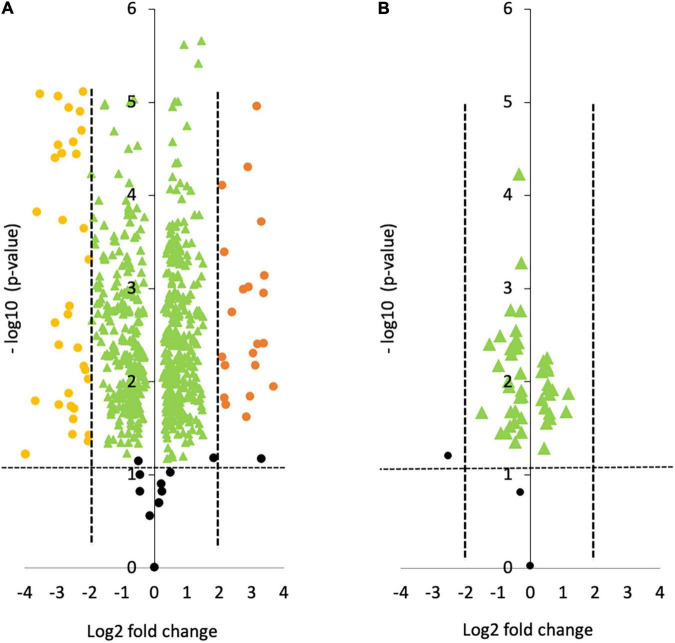
Volcano plots representing the pairwise comparison of **(A)** Oval vs. Fusiform (OF); **(B)** Triradiate vs. Fusiform (TF). The volcano plots show a change in protein expression (*t*-test *p*-value) between **(A)** oval and fusiform cells, **(B)** triradiate and fusiform cells. Fusiform cells are considered as the reference. Orange and yellow dots represent up-regulated and down-regulated proteins at a log fold change upper or lower than 2, respectively (*p*-value ≤ 0.05). Green dots represent protein that has a fold change comprised between –2 and 2. Black dots represent the non-differentially expressed proteins.

The intracellular protein profile from the oval morphotype (O) was compared to the one of the fusiform (F) morphotypes used as a reference (O vs. F, noted OF). When comparing OF, a total of 691 proteins were identified. As represented in the volcano plot shown in Figure 2A, 61 proteins were statistically differentially produced at a log fold change greater than –2 or 2 in the O morphotype compared to the F morphotype. Among them, 38 proteins were down-regulated whereas 23 were up-regulated respectively. In contrast, when comparing the Triradiate (T) morphotype to the F one (T vs. F noted TF), only 50 proteins were differentially produced with a log fold change comprised between –2 and 2 (Figure 2B). No proteins were up or down regulated at a log fold change higher than –2 or 2 in the TF comparison. These results suggest that there is not much difference in the intracellular protein profile of TF.

**FIGURE 3:**
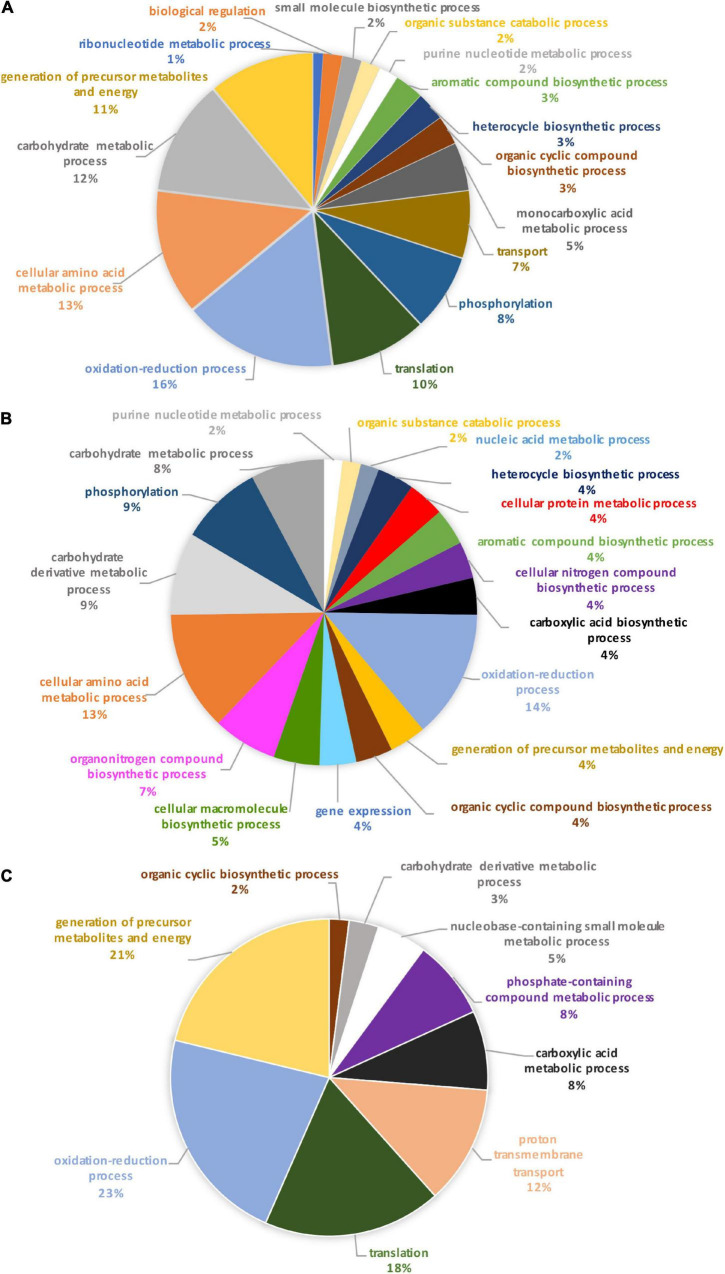
Pie charts representing the biological processes, which are alliterated in the OF pairwise comparison. **(A)** Overall biological processes associated with the overall differentially expressed proteins in the oval morphotype when compared to the fusiform one. **(B)** Biological processes which are associated with the up-regulated proteins. **(C)** Biological processes associated with down-regulated proteins.

Identification of these differential proteins was performed using Blast2GO. This software allows the characterization and classification of proteins into biological processes, molecular and cellular functions according to GO annotation. At this stage, non-significantly differentially produced proteins (*p*-value > 0.05) identified from the proteomic label-free analysis were removed (11 proteins). Thus, the Blast2GO analysis was run on 680 proteins resulting from the OF pairwise comparison and 48 proteins for the TF pairwise comparison (*p*-value ≤ 0.05). From this analysis, about 84% of the intracellular proteins from the OF and 94% of intracellular proteins from the TF pairwise comparison have been annotated with GO terms thus corresponding to a total of 569 and 45 proteins respectively. Repartition of the identified proteins was performed based on their involvement in specific biological processes. A Pie chart representing the OF biological processes (Figure 3A) showed that oxidation-reduction (16%), cellular amino acid (13%), carbohydrate (12%), translation (10%), and energy (11%) processes represent overall 62% of the differentially produced proteins. Transport and phosphorylation processes represented 7 and 8%, respectively. In contrast, purine nucleotide metabolic pathway and ribonucleotide metabolic pathways represent 2 and 1% respectively. Other biological processes such as small biosynthetic molecules, heterocycle, cyclic compounds, and monocarboxylic acid metabolic pathways represent less than 5% for each category. A GO category dedicated to biological regulation also emerged at 2%.

To further understand the specific regulation between *P. tricornutum* morphotype, up- and down-regulated proteins were subjected to GO analysis separately. In brief, 55% of the 569 annotated proteins with a GO term are up-regulated in the O morphotype and 45% are down-regulated in comparison to the F morphotype. Among the up-regulated proteins (Figure 3B), 13% are related to the cellular amino acid process; 9% correspond to the carbohydrate derivative metabolic process, 9% to phosphorylation, and 8% to the carbohydrate metabolic process. Purine nucleotide metabolic and nucleic acid metabolic processes represent 2% respectively whereas cellular protein metabolic and gene expression processes represent 4% (Figure 3B). In contrast, proteins associated with the synthesis of precursor metabolites and to energy processes (21%), to translation (18%), proton transmembrane transport (12%), carboxylic acid metabolism (8%) and phosphate-containing compound metabolism (8%) processes are down-regulated in O cells (Figure 3C). Particular attention was paid to the oxidation-reduction GO term as proteins involved in this biological process are either up-regulated (14%) or down-regulated (23%) in the O morphotype. Similarly, proteins involved in the carbohydrate metabolism are either up- (9%) or down-regulated (3%). These results suggest a fine-tune regulation of proteins involved in these specific biological processes (Figures 3B,C).

As previously described in the OF volcano plot (Figure 2), an abundance of 61 proteins changed at least twofold with *p-*values lower than 0.05. Among the 23 up-regulated proteins, 13 exhibits at least one known function and are mainly involved in the oxidation-reduction process like the cytochrome P450, the SDR family oxidoreductase, alkene reductase, and hydroxylamine reductase (Table 2). Ten proteins from this subset do not display either description or biological function (Table 2). Similarly, among the 38 down-regulated proteins, 23 of them exhibited a biological function in relation to cellular adhesion, transport, and catalytic activity (Table 3), whereas the functions of 15 proteins were not elucidated yet as they could not be related to any known biological function.

**TABLE 2:** Biological process in which oval morphotype up-regulated proteins are involved.

SeqName	ProteinName	Description	Biological function
Phatr3_Jdraft1820	B7S4B2	Alcohol dehydrogenase	Glycolysis
Phatr3_J18911	B7FTW1	Aspartate–ammonia ligase	Acid amino synthesis
Phatr3_EG02188	B7FPT2	Protein *S*-acyltransferase	Protein degradation
Phatr3_J34976	B7FX80	Glutathione *S*-transferase mu 3	Cellular response to chemical stimulus
Phatr3_J43466	B7FSB5	Cytochrome P450	Oxidation-reduction process
Phatr3_J45046	B7FWA5	CBS domain-containing protein	Metabolic and cellular process
Phatr3_J45621	B7FYB0	NO-inducible flavohemoprotein	Response to nitrosative stress
Phatr3_J35939	B7FZX8	SDR family oxidoreductase	Oxidation-reduction process
Phatr3_J49119	B7G9J7	Alpha/beta hydrolase	Catalytic and hydrolase activity
Phatr3_J37667	B7G3E9	SDR family oxidoreductase	NA
Phatr3_J49937	B7GCD4	Predicted protein	NA
Phatr3_EG02230	B7G9U5	Predicted protein	NA
Phatr3_J46597	B7G1T2	Predicted protein	NA
Phatr3_J50914	B7FZJ4	Alkene reductase	Oxidation-reduction process
Phatr3_EG02330	B7G2D1	Predicted protein	NA
Phatr3_J33876	B7FU42	Predicted protein	NA
Phatr3_J15393	B7G884	NAD(P)H:quinone oxidoreductase, type IV	Oxidation-reduction process
Phatr3_J47823	B7G516	Predicted protein	NA
Phatr3_J44546	B7FUG6	Aldo/keto reductase	Oxidation-reduction process
Phatr3_J44092	B5Y5B5	Predicted protein	NA
Phatr3_Jdraft1693	B7S462	Predicted protein	NA
Phatr3_J12416	B7FZQ2	Hydroxylamine reductase	Oxidation-reduction process
Phatr3_J47840	B7G535	Predicted protein	NA

*Only proteins with a log fold change greater than 2 are presented. NA: no biological process was associated with the Blast2GO software.*

**TABLE 3:** Biological process in which oval morphotype down-regulated proteins are involved.

SeqName	ProteinName	Description	Biological function
Phatr3_EG02655	B7FXJ4	Fasciclin domain-containing protein	Cellular adhesion
Phatr3_J45403	B7FXK5	Predicted protein	NA
Phatr3_J44526	B7FUE7	Predicted protein	Carbonic anhydrase alpha enzyme
Phatr3_J50592	B7GEL9	Predicted protein	Cell wall/membrane/ envelope biogenesis
Phatr3_J46400	B7G133	Predicted protein	Catalytic activity
Phatr3_J49202	B7G9T6	Predicted protein	NA
Phatr3_J49297	B7GA49	Predicted protein	NA
Phatr3_J45402	B7FXK4	Predicted protein	NA
Phatr3_J48730	B7G866	Predicted protein	NA
Phatr3_J48704	B7G7F7	Methyltransferase domain-containing protein	Methyltransferase activity
Phatr3_J49567	B7GB24	Predicted protein	DNA-binding transcription factor activity
Phatr3_J45464	B7FXS7	Predicted protein	NA
Phatr3_EG02527	B5Y460	V-type H(+)-translocating pyrophosphatase	Inorganic diphosphatase activity
Phatr3_EG02354	B7FVR9	Predicted protein	NA
Phatr3_J17519	B7FPK3	40S ribosomal protein IP6	Structural constituent of ribosome
Phatr3_J48383	B7G6Y2	Predicted protein	NA
Phatr3_J54686	B7G2A6	Predicted protein	Catalytic activity
Phatr3_EG02167	B7FQE7	Predicted protein	NA
Phatr3_EG02265	B7G6X2	Predicted protein	3′,5′-cyclic-nucleotide phosphodiesterase activity
Phatr3_J46046	B7FZV5	Predicted protein	NA
Phatr3_J49296	B7GA48	Predicted protein	NA
Phatr3_J41518	B7GEF5	Predicted protein	Methyltransferase activity
Phatr3_J50019	B7GCM8	Predicted protein	NA
Phatr3_J47667	B7G4H1	Predicted protein	Sodium-dependent phosphate transmembrane transporter activity
Phatr3_J47412	B7G3A5	Predicted protein	Nucleic acid binding
Phatr3_J48827	B7G8I4	Predicted protein	NA
Phatr3_J46547	B7G1L3	Predicted protein	Serine-type endopeptidase activity
Phatr3_J40158	B7GAM6	Predicted protein	Nucleic acid binding
Phatr3_J54642	B5Y3R0	Transitional endoplasmic reticulum ATPase	ATP binding
Phatr3_J12989	B7G1S8	NarL family transcriptional regulator	Catalytic activity
Phatr3_J49215	B7G9V1	Predicted protein	NA
Phatr3_J52619	B7GBV6	Purine permease	Transmembrane transporter activity
PHATRDRAFT_55198	B7GE39	STT3 subunit-like protein	Protein amino acid glycosylation
PHATR_10209	B5Y4T4	Coatomer subunit gamma	Vesicle transport intracellular transport
PHATRDRAFT_45808	B7FYR4	Predicted protein	NA

*Only proteins with a log-fold changed lower than 2 are presented.*

*NA, no biological process was associated with the Blast2GO software.*

**FIGURE 4:**
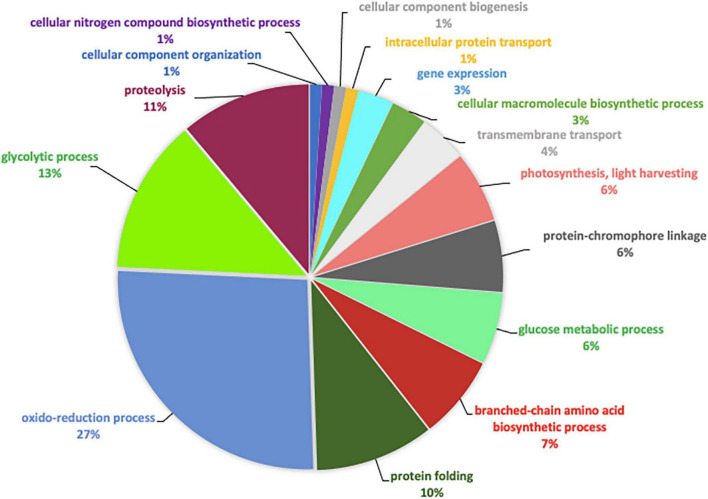
Pie chart representing the biological processes, which are alliterated in the TF pairwise comparison.

When comparing the pie chart representing the TF biological process pairwise comparison (Figure 4), we have shown that the oxidation-reduction biological process represents 27%, the glycolytic process 13%, the proteolysis 11%, the protein folding 10%, thus covering above 61% of the differentially produced proteins. In addition, proteins involved in the biosynthetic process of the branched amino acids, in the glucose metabolic process, in photosynthesis, and in light-harvesting accounted for 6% each. Other GO biological processes like transmembrane and intracellular transport, gene expression, cellular macromolecule biosynthetic process, and cellular component biogenesis were also found.

### Specific Metabolic Pathways Are Involved in the Oval and Triradiate Morphotypes

**FIGURE 5:**
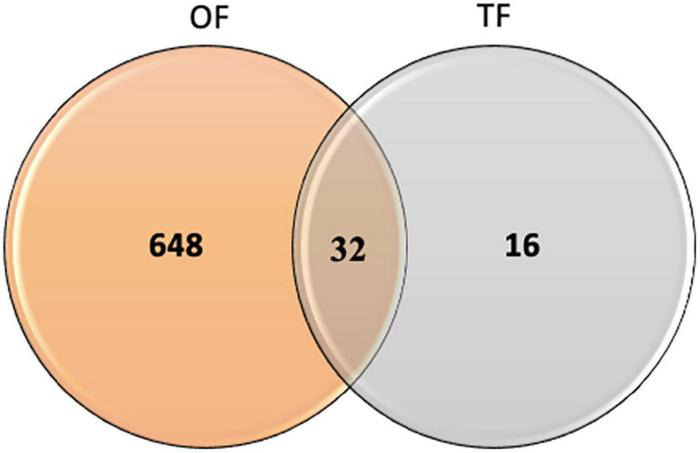
Venn diagram displaying the specific features of proteins identified in the Oval vs. Fusiform (OF) and Triradiate vs. Fusiform (TF) pairwise comparisons. Thirty-two proteins were overlapping between the OF and TF comparisons. Six hundred and forty-eight and 16 proteins were specifically expressed in oval and triradiate morphotypes respectively. Venn diagram analysis was achieved using the 680 proteins subset identified in the OF pairwise comparison and the 48 proteins identified for the TF pairwise comparison (*p*-value ≤ 0.05).

**FIGURE 6:**
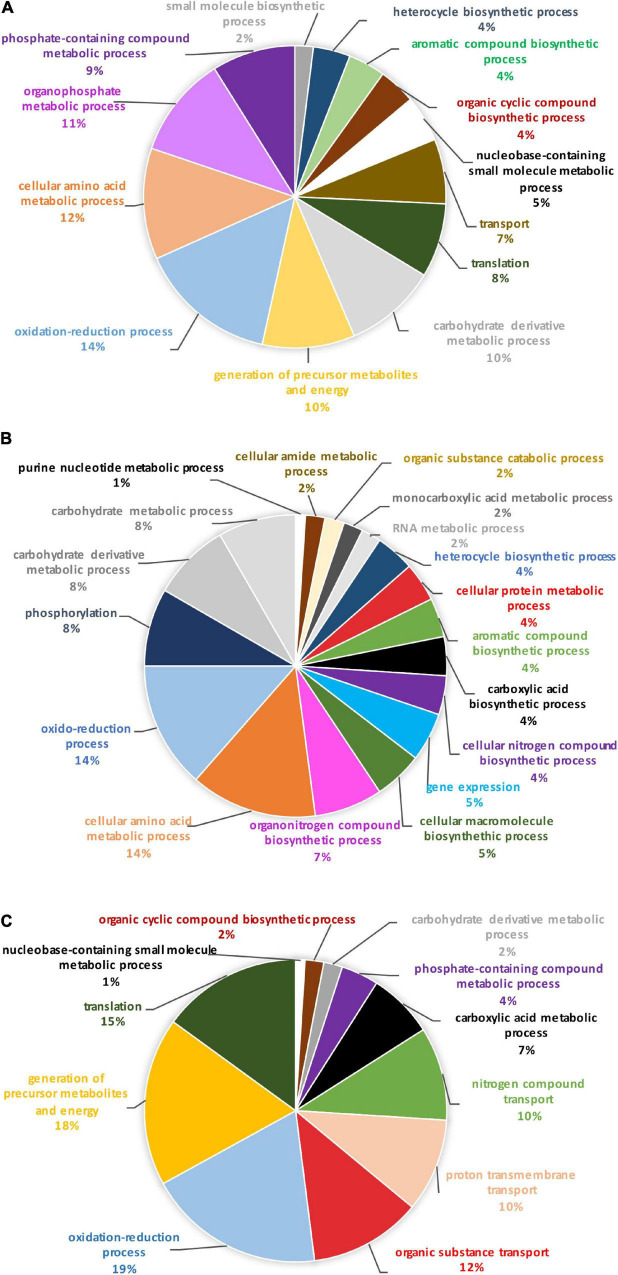
Pie chart representing the biological process in which the 648 proteins, unique to the oval morphotype are involved. **(A)** Overall biological processes associated with the overall specific expressed proteins in the oval morphotype. **(B)** Biological processes which are associated with the up-regulated proteins. **(C)** Biological processes associated with down-regulated proteins.

**FIGURE 7:**
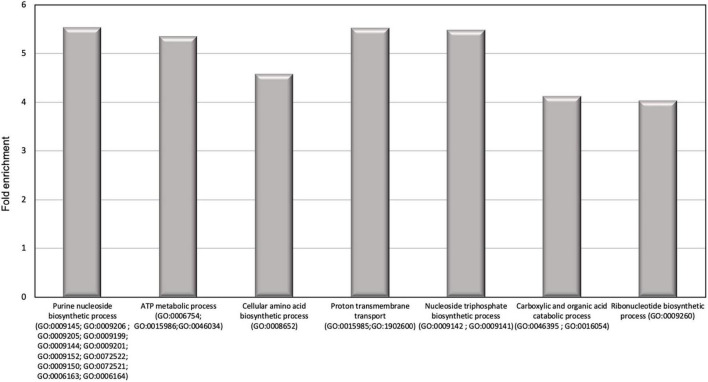
Results of the overrepresentation test (Panther) of the 648 proteins specifically expressed in oval morphotype. Only proteins families with a fold enrichment upper than 4 are represented.

In order to identify the proteins, which can be specifically assigned to either the oval or triradiate morphotypes, intersection analysis was performed and presented by the Venn diagram in Figure 5. This figure demonstrates that 32 proteins were identified at the intersection of the OF and TF pairwise comparisons, thus being specific to fusiform morphotype (Supplementary Data 1). A large portion of these 32 proteins identified is related to the oxido-reduction process (31%), followed by protein involved in proteolysis (16%) and protein folding (6%). In total, these three categories represented up to 53% of the biological processes. Other categories such as glycolytic and glucose metabolic processes represent 6% of the biological processes identified in the F morphotype (Supplementary Figure 2 and Supplementary Data 1). Sixteen proteins were identified to be specific to the T morphotype (Figure 5). Among these T-specific proteins, some are belonging to protein folding, photosynthesis/light-harvesting, oxido-reduction process, proteolysis (Supplementary Figure 3 and Supplementary Data 2). As far as the Oval morphotype is concerned, 648 proteins were identified to be specifically produced in the O morphotype in the intersection analysis (Figure 5). Among these 648 specific proteins, Blast2GO analysis was able to annotate and attribute GO biological processes to 544 proteins. Cellular amino acid metabolic process (12%), organophosphate metabolic process (11%), oxido-reduction process (14%), generation of precursor metabolites and energy (10%), phosphate-containing compound (11%), carbohydrate derivative process (10%) and translation (8%) are the most represented GO biological processes in O morphotype (Figure 6A). More precisely, our results show that phosphorylation (8%), cellular amino acid metabolic process (14%), carbohydrate (8%), and organonitrogen compound biosynthetic process (7%) are specifically up-regulated in the O cells (Figure 6B) whereas processes such as translation (15%), generation of precursor metabolites and energy (18%), organic substance transport (12%) and proton transmembrane transport (10%) are down-regulated (Figure 6C). To get information on differentially produced proteins involved in the specific biological pathway, analysis of the protein families was carried out with PANTHER. This analysis revealed that some proteins families are significantly overrepresented in the O cells compared to the other morphotypes. Many of the GO terms that were overrepresented were related to the purine nucleoside metabolism with a fold enrichment close to 6, to the ATP metabolic process (fold enrichment 5.5), and to the cellular amino acid biosynthetic process (fold enrichment 4.5) (Figure 7). The overrepresentation of these terms could indicate an expanded network that synthesizes specific metabolites in O cells.

**FIGURE 8:**
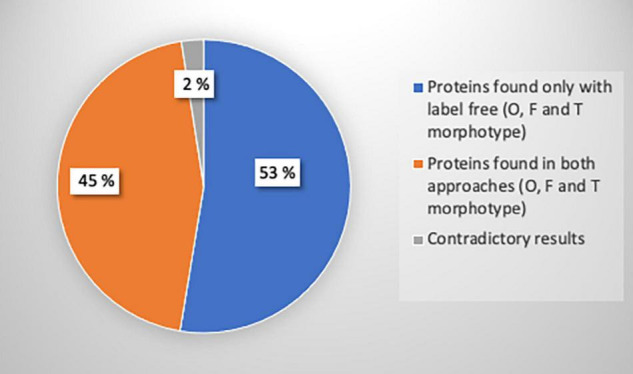
Pie chart representing the number of proteins identified with iTRAQ^®^, label-free or both methods, and the proportion of contradictory results. A total of 696 proteins were identified with label-free analysis (*p*-value ≤ 0.05): 648 proteins for oval, 32 proteins for fusiform, and 16 proteins for triradiate morphotypes. A total of 330 proteins were identified with iTRAQ^®^ analysis. Among these proteins, 17 proteins representing 2% of iTRAQ^®^ identified proteins were differentially regulated between both approaches.

To confirm previous results obtained with the label-free proteomic approach, we decided to perform quantitative proteomic analysis using iTRAQ^®^ (for isobaric tags for relative and absolute quantification) labeling. In this experiment, the intracellular proteins extracted from the O, the T, and the F morphotypes were tagged respectively with iTRAQ^®^ reagents 115, 116, and 117. Differential protein production was defined as an iTRAQ^®^ ratio between the O or T morphotype using the F morphotype as a reference and ratios with a fold change higher than 1.2 were considered (Supplementary Data 3). The outcome of the iTRAQ^®^ analysis allowed identifying 330 proteins, which are strictly identical to the subset of the 696 proteins identified with the label-free approach, thus corresponding to above 44% of the proteome (Figure 8). Only 2% of contradictory results were observed on a total of 330 common proteins (Figure 8). Results showing the differentially expressed proteins with the iTRAQ^®^ methods and label-free analysis are presented in Supplementary Figure 3. Overall, the iTRAQ^®^ results are consistent with those of the label-free analyses regarding the quantification of the differentially produced proteins. These results reveal that similarly to the label-free analysis, the proteasome, nucleoside metabolic process, photosynthesis, carbohydrate, and cellular amino acid biosynthesis were overrepresented highlighting a good correlation and the robustness of the data resulting from the label-free and iTRAQ^®^ analyses. However, the label-free approach allowed us to identify more proteins, so we decided to use this method for the analysis of the secretomes.

### Differences in Extracellular Secreted Protein Productions Exist Between the Three Pt3 *Phaeodactylum tricornutum* Morphotypes

Proteins that are secreted in the culture media by the diatom cells constitute the secretome. Such proteins may play important roles in cell migration, cell signaling, defense, and communication. These proteins can also be degraded into amino acids and serve as a source of nitrogen and/or carbon.

**FIGURE 9:**
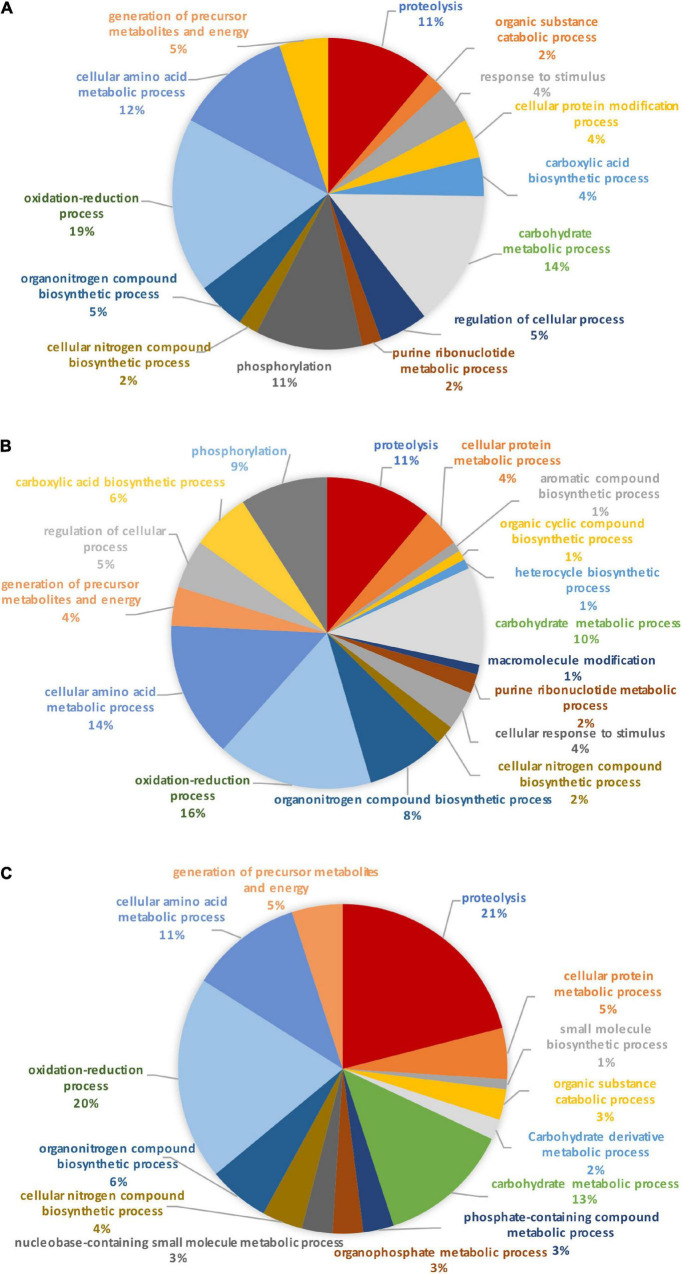
Pie charts representing the biological process in which **(A)** fusiform, **(B)** triradiate, and **(C)** oval morphotype secreted proteins are involved. Pie charts are representative of 5 biological replicates. **(A)** Biological processes of the 275 proteins secreted in fusiform morphotype. **(B)** Biological processes of the 231 proteins secreted in triradiate morphotype. **(C)** Biological processes of the 385 proteins secreted in oval morphotype.

In order to further characterize the *P. tricornutum* morphotypes, we decided to identify the secretome of the previous enriched cultures. Overall, 949 secreted proteins were identified in the culture media of the three morphotypes with the label-free analysis. As previously described, Blast2GO software was used to attribute GO annotation and determine the biological functions in which these proteins are involved. From this analysis, above 87.5% of the secreted proteins have been annotated with GO terms thus corresponding to a total of 831 proteins (375 proteins for O, 207 for T, and 251 for F secreted proteins). Repartition of the identified proteins based on their biological process was accomplished. Pie chart representing the F biological process (Figure 9A) shows oxidation-reduction (19%), cellular amino acid (12%), carbohydrate (14%), phosphorylation, and proteolysis (11%) processes represent overall 56% of the differentially produced proteins. The response to stimulus and regulation of cellular process represent 4 and 5% while purine nucleotide metabolic pathway and cellular nitrogen biosynthetic pathway represent 2% of the biological process. Concerning the proteins which are secreted in the culture media of the T cells, it appears that the biological processes in which they are involved are relatively similar to the ones of F secreted proteins (Figure 9B). Proteins involved in oxidation-reduction processes represent up to 16%, followed by protein involved in cellular amino acids (14%), carbohydrates (10%), phosphorylation (9%), and proteolysis (11%), thus representing overall 60% of the differentially produced proteins. Interestingly, the biological process associated with macromolecule modification (1%), aromatic compound biosynthetic pathway (1%) appears to be specifically present in the triradiate secreted proteins. As far as the secreted proteins from the O morphotype are concerned, biological processes such as proteolysis (21%) and cellular nitrogen compound biosynthetic pathway (4%) are increased in comparison to the two other morphotypes (Figure 9C).

**FIGURE 10:**
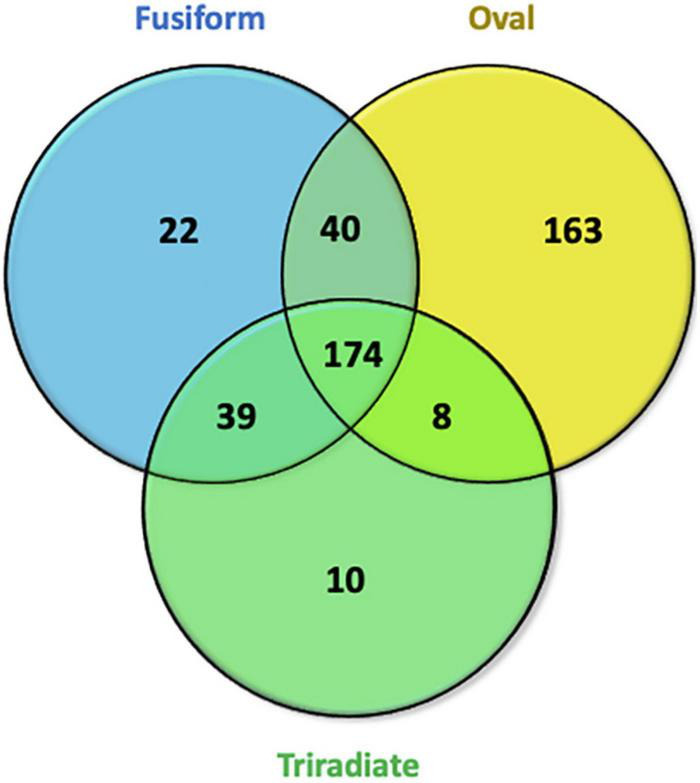
Venn diagrams displaying overlaps of secreted proteins identified in Oval, Fusiform, and Triradiate cells. In the total proteins, 174 secreted proteins were identified in the three groups. Thirty-nine proteins were present in fusiform and triradiate cells, 40 proteins were present in fusiform and oval cells, 8 proteins were present in triradiate and oval cells. 22, 10, and 163 appear to be specific to fusiform, triradiate, and oval cells, respectively. Venn diagrams are representative of 5 biological replicates for each morphotype.

**FIGURE 11:**
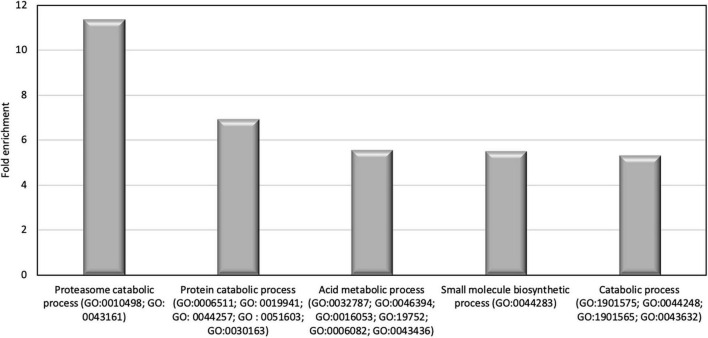
Results of the overrepresentation test (Panther) of the 163 proteins found in the oval cell culture medium. Only proteins families with a fold enrichment upper than 4 are represented.

To go further in the characterization of *P. tricornutum* secretomes, an intersection analysis was run in order to identify the proteins, which can be specifically attributed to either the oval, fusiform, or triradiate secretomes. The resulting Venn diagram presented in Figure 10 demonstrated that among the identified secreted proteins, 22, 10, and 163 secreted proteins were specific to the F, T, and O morphotypes, respectively (Supplementary Data 4). Interestingly, this result clearly confirmed previous observation (Ovide et al., 2018; Song et al., 2020; Galas et al., 2021) that the O cells are secreted more proteins than the two others morphotypes. Again, the F and T secretomes appeared to be very similar (Figure 10). Analysis of the protein families by PANTHER highlighted that proteasome catabolism (fold enrichment 11), protein catabolism (fold enrichment 7), and acid catabolic process (fold enrichment 6) are significantly overrepresented in the medium of O cells compared to the other morphotypes (Figure 11). No specific enrichment was observed for secreted proteins of F and T cells probably due to the fact that the number of specific proteins for both morphotypes was relatively low (22 and 10, respectively).

### The Secretomes of the Three Morphotypes Are Particularly Enriched in Glycoproteins

In order to confirm the proteins identification in the secretomes of the different morphotypes, we look for the presence of a putative signal peptide and *N*-glycosylation consensus sites that are characteristics of proteins intended to travel through the secretory pathway and to be secreted in the culture medium. The use of the SignalP online tool allows us to predict the presence of a signal peptide for more than 47.5% of proteins in the F secretome, almost 40% of proteins in the T one, and 50% in the O one. It is to note that only predictions of signal peptides with a probability greater than 0.5 were considered (Supplementary Data 5, excel sheet 1). Indications regarding the position of the signal peptide are also available (Supplementary Data 5, excel sheet 1). For comparison, prediction of the signal peptide of the intracellular proteins identified for each morphotype has been looked for, showing an average of 26.5% predicted signal peptide in the overall intracellular proteins. With regards to the *N*-glycosylation predictions that were run using the NetNGlyc tool, 67% of the F secreted proteins, 64.5% of the T secreted proteins, and finally, 71.3% of the O secreted proteins were predicted to bear *N*-glycosylation sites, respectively. This represents an average of 2.67 *N*-glycosylation sites per secreted protein independently of the morphotypes (Supplementary Data 5, excel sheet 1).

### Analysis of the Putative Evolutive Origin of the Secreted Proteins

In order to determine the origin of the secreted proteins that are secreted by the different morphotypes of *P. tricornutum* Pt3 strain, we compared the dataset from this study to the one reported in Dorrell et al., 2021 (Supplementary Data 5, excel sheets 2–5). The comparison allows us to determine that among the 274 secreted proteins that were identified in the F morphotype, only 22 were also present in the Dorrell et al. (2021) dataset. Such subset is enriched mainly in proteins originating from bacteria (8) representing 36.4%, alveolates (6) representing 27.2%, and dinoflagellates (4) accounting for 18.2% (Supplementary Data 5, excel sheets 2, 5). A similar trend was observed for 23 out of 230 proteins identified in the T morphotype. Among these proteins, 7 originated from bacteria (30%), 6 from alveolates (26%), and finally 5 from dinoflagellates (21.7%) (Supplementary Data 5, excel sheets 3, 5). As far as the secreted proteins from the O morphotype are concerned, only 13 out of 384 were common with previous data from Dorrell et al. (2021). Among the 13 proteins, 5 are originated from bacteria, thus representing 38.4% and 3 originated from dinoflagellates (23%), the others coming from alveolates (2), haptophytes (2), and Rhizaria (1) lineages (Supplementary Data 5, excel sheets 4, 5). Finally, the list of 13 proteins from the overall O secretome was crossed with the 163 proteins that are specific to the O morphotype. It came out from this comparison that only 3 were specific to the O morphotype: the protein B7G0Q2 (Phatr3_J20677) and the protein B7FZM8 (Phatr3_J35518) originated from bacteria whereas the protein B5Y4E4 (Phatr3_J33120) derived from Rhizaria. The percentage of secreted proteins originating from bacteria displays above 35% of the secretome independently of the morphotype. This number is below the 49% observed in the overall genome of *P. tricornutum* for which bacteria were reported to be responsible for nearly half of the gene transfers (Dorrell et al., 2021).

### Comparative Analysis of the Proteome Versus Transcriptome

In order to evaluate the relationship between previous transcriptomic data reported on the Pt3 morphotypes and the current proteomic data, we analyzed and compared the proteome of the O cells to the transcriptomes reporting in Ovide et al. (2018) that compared O vs. F (OF) and O vs. T (OT). Among the 648 intracellular proteins that were specifically identified in the O proteomic data, 35.65% were also differentially expressed in the transcriptome data comparing OT and OF. A similar comparison was made for the 163 secreted proteins that have been identified in the secretome of the O cells and above 32.7% were also differentially expressed in the transcriptome dataset (Supplementary Data 6).

## Discussion

The diatom *P. tricornutum* exists under three distinct major morphotypes, fusiform, triradiate, and oval cells that can be observed mostly depending on the environmental conditions (Tesson et al., 2009). The fusiform morphotype is considered the most common one (De Martino et al., 2007, 2011; Ovide et al., 2018). As a consequence, mainly research performed on *P. tricornutum* fusiform morphotype is widely available (Xie et al., 2015; Bai et al., 2016; Longworth et al., 2016). However, a transcriptome-wide characterization of the three morphotypes originating from the Pt3 strain has recently been performed revealing differences in transcriptomic regulation between morphotypes (Ovide et al., 2018). Moreover, recent biochemical and imaging analyses revealed that the oval morphotype is synthesizing more proteins (Song et al., 2020) and is secreting proteins more rapidly as compared to the other morphotypes (Galas et al., 2021). However, so far, no proteomic studies of the three morphotypes have been carried out. In the present work, a total of 728 intracellular proteins of *P. tricornutum* were identified. Pairwise comparisons showed that fusiform and triradiate morphotypes were very similar as only the expression of 48 intracellular proteins were differentially regulated between these two morphotypes whereas 680 intracellular proteins were differentially regulated when comparing the oval morphotype versus the fusiform one. This result is consistent with previous reports that reported fusiform and triradiate morphotypes as similar morphotypes (less than 1% of the transcriptomes were significantly differentially expressed, Ovide et al., 2018) whereas the oval cells were described to be more active metabolically (Bartual et al., 2008; Ovide et al., 2018; Song et al., 2020). Interestingly, in our study, 648 intracellular proteins appeared to be oval specific confirming that the oval morphotype is more metabolically active.

Biological processes such as purine (ribo)nucleoside metabolic pathway, carbohydrate, and cellular amino acid biosynthesis are overrepresented in the oval cells while representing only 2% of the overall intracellular proteins. These findings indicated that the oval morphotype promotes nucleotide, carbohydrate, and amino acid biosynthesis. Such an increase has been previously reported by Ovide et al. (2018) where 68% of genes involved in the primary metabolism pathway (glycolysis, nucleotide, etc.) were increased in the OF pairwise comparison. The increased production of proteins responsible for various biosynthetic processes suggests that the oval cells are extremely responsive to their environment. It has been hypothesized that the oval cells are more resistant to stresses as this morphotype seems to be favored under extreme conditions such as low temperature, the presence of bacteria, or low salinity (De Martino et al., 2011; Buhmann et al., 2016). In our study, upregulation (>2-fold) of proteins such as B7FX80, a glutathione S-transferase mu 3 implicated in the cellular response to stimulus, and B7FYB0, a NO-inducible flavohemoprotein involved in response to nitrosative stress, as well as proteins involved in the oxidative process, was consistent with this hypothesis and reflects the specific regulation of stress-responsive proteins. However, we should keep in mind that oval cells were cultured in 10 % seawater in order to reach an oval cells enrichment of 98% but also because it was not possible to maintain an enriched culture in oval morphotype using 100% seawater media (Ovide et al., 2018). The reverse was not possible with fusiform and triradiate cells in 10% seawater as most cells switched into oval cells when maintained in such culture conditions. As a consequence, we hypothesize that the oval cells present a metabolism specifically adapted to environment variables. Oval cells may contain specific proteins involved in repair mechanisms in contrast to the fusiform and triradiate cells allowing the oval cells to survive when conditions become unfavorable. The presence of such proteins in oval cells may explain the interconversion of fusiform or triradiate morphotype into this more resistant morphotype.

This agrees with the fact that diatoms have developed defense mechanisms to overcome unfriendly environments (Falciatore et al., 2020). In this study, we noticed the presence of proteins such as B7FPT2 identified as a palmitoyltransferase, B7FUR0, and B7FYUX1 involved in terpenoid biosynthesis in the oval cells. This may suggest the production of defense molecules in this morphotype. Similarly, earlier studies demonstrated that *P. tricornutum* was able to synthesized molecules known to exert antimicrobial activity such as terpenoids (α-1, 8-cineole, α-pinene, limonene; Prestegard et al., 2015), palmitoleic acid (Desbois et al., 2009), or reactive oxygen species (Buhmann et al., 2016) in higher amounts in oval cells. Altogether, it appears likely that oval cells synthesize molecules specifically involved in defense pathways against predators or pathogen organisms. Interestingly, the two glyceraldehyde-3-phosphate dehydrogenases (B7G5Q1; B7G6K6), the oxygen-evolving enhancer protein B7F296 and the phosphoribulokinase B5Y5F0 of which abundances were reported to increase in fusiform cells after 4 days of darkness (Bai et al., 2016), are more abundant in Pt3 oval cells in comparison to Pt3 fusiform cells. As a consequence, we hypothesize that oval cells naturally synthesized more proteins related to dark conditions compared to fusiform cells. To challenge this hypothesis, we compared the list of genes encoding proteins specific to the oval cells to the 104 genes with robust diel oscillating expression reported in Annunziata et al. (2019). The expression of the 104 genes was studied using a 16h:8h light: dark cycle as the one that was used in this study. However, once should keep in mind that the media used in the two studies to grow the cells are not exactly the same (f/2 Guillard media in Annunziata et al., 2019 and Conway in the present study) and that the harvest of the diatom cells in this study were done few hours after illumination. From that comparison, only 11 were found in the intracellular proteins of the oval cells, none of them being photoreceptors. Among the 11 candidates, 7 are implicated in the metabolism (Pds1, Gapc, Psy1, Gsat, Zep3, CaThioredoxin, and Zds), 2 belong to the cell cycle (FtsZ and Pcna) and the two last ones were transcription factors (HSF4.2j and HSF4.7a). This might also be correlated with the fact that fusiform cells have a planktonic lifestyle considering that oval cells are preferentially benthic morphotypes (De Martino et al., 2007; Stanley and Callow, 2007; Willis et al., 2013). Therefore, the difference in lifestyle exposed the morphotypes to light variation with the fusiform cells benefiting from a higher exposure. Moreover, it is to note that most of these proteins are involved in the carotenoid biosynthesis pathway (Pds, Psy, Zep, Zds) (Bertrand, 2010; Scarsini et al., 2020) and in photosynthesis (Gsat; Schoefs and Bertrand, 2005).

When comparing previous transcriptomic data (Ovide et al., 2018) and the proteomic datasets described here, some discrepancies and only 1/3 of positive correlation were observed. Such differences could be related to a fine-tuning gene expression that can be due to allele-specific expression (ASE). Such phenomena have been recently described in *P. tricornutum* (Hoguin et al., 2021). Such ASE genes were enriched in genes involved in catabolism processes including proteasome subunits proteins, and autophagy, in intracellular protein transport, exocytosis, and endocytosis (Hoguin et al., 2021) that correspond to pathways identified in this study. Moreover, it would also be interesting to study gene alternative splicing as it was reported previously that extensive alternative splicing was involved in the regulation of gene expression in response to nutrient starvation, suggesting that *P. tricornutum* used it to cope with environmental changes (Rastogi et al., 2018).

However, it should not be forgotten that in our study GO terms were attributed for only 608 proteins from a total of 728 intracellular proteins. This means that even 12 years after the genome of *P. tricornutum* was sequenced (Bowler et al., 2008), there is at least 16 % of predicted proteins of which the biological function remains unanswered. In the future, a lot of work remains to be performed in order to identify the physiological role of those proteins in *P. tricornutum*. Moreover, it would be interesting to determine how many of the differentially accumulated proteins are encoded by genes that show SNPs. Indeed, Rastogi et al. (2020) recently revealed the global genetic polymorphism, structure, and functional diversity of ten accessions strains of *P. tricornutum* showing high differences in SNPs between ecotypes. Therefore, it would be interesting in future studies to analyze if proteins specific to the oval morphotype are more likely to contain SNPs. In this study, it was chosen to work with enriched cultures of oval, triradiate, and fusiform morphotypes issued from the same Pt3 strain and compared their specific proteomes and secretomes. It would be interesting in future studies to compare the proteome of Pt3 that is naturally rich in oval cells, one of the Pt1 (naturally rich in fusiform cells), and one of the Pt8 (rich in triradiate) and evaluate whether specific proteome signature can be identified for each morphotype regardless of the original strain.

To go further in the characterization of the *P. tricornutum* morphotype, we also expanded the analysis to the identification of the secreted proteins of each morphotype. It is to note that during the preparation of the samples, the culture medium was desalted and concentrated using a 3K MWCO Concentrator, thus, removing all peptides below 3 kDa. It will be interesting in a future study to characterize such a population of peptides that could be interesting small bioactive peptides differentially expressed between morphotypes.

As for the intracellular proteomic analysis, our results show that the number of secreted proteins was higher in the oval cells with a total of 385 proteins whereas in fusiform and triradiate cells several 275 and 231 secreted proteins were totalized, respectively. Among those proteins, 40 to 50% possess a predicted signal peptide with a probability greater than 0.5 while only 13.3% (1,629 proteins out of 12,233) to 15% (1,831 proteins out of 12,179) were reported in the entire proteomes of *P. tricornutum* in the study of Ait-Mohamed et al. (2020) and Rastogi et al. (2020), respectively. Moreover, in this study, as expected, the intracellular proteins present only 26.5% of the predicted signal peptides on average. The Venn diagram identified 163 proteins that were specific for the oval cells whereas only 22 and 10 secreted proteins were specific for fusiform and triradiate morphotypes, respectively. This result, in agreement with previous reports, demonstrates that in the oval morphotype, more proteins are secreted (Ovide et al., 2018; Song et al., 2020). This higher number of secreted proteins in the oval cells can be related to the excretion of exopolymeric substances that favor cell adhesion and colony formation specific to the oval morphotype (Stanley and Callow, 2007; Willis et al., 2013). The difference in adhesion of *P. tricornutum* cells was reported to be dependent on the composition of the various types of EPS produced (Stanley and Callow, 2007), which can be explained by differences in the proportion of monosaccharides, chain terminal saccharide and the degree of sulphation. For example, salinity changes resulted in an increase of carbohydrate production, with enrichment of highly branched/substituted and terminal rhamnose, xylose, and fucose as well as *O*-methylated sugars, uronic acids, and sulfate (Abdullahi et al., 2006).

Recently, Erdene-Ochir et al. (2019) reported that the protein B7G4A0, also known as HASP1 protein, is the most abundant protein secreted into the culture medium of *P. tricornutum*. Interestingly, this finding is also supported by our results as we noted the presence of B7G4A0 in the culture media of the fusiform and triradiate cells. By contrast, we do not report the presence of this protein in the culture medium of the oval cells. In addition, our results also confirmed that the proteins B7FSH1 and B7G259 reported being the third and the fourth most secreted proteins reported by Erdene-Ochir et al. (2019), are also detected in the culture media of the three morphotypes. In addition, the proteins B5Y3F2 and B7GBF3, which were reported to be abundant in the culture media of *P. tricornutum*, were only detected in the oval and triradiate culture media in our conditions. Such results are of particular interest as the HASP1 gene promoter for example has been used to improve the production of secreted recombinant proteins by *P. tricornutum* (Erdene-Ochir et al., 2019). In addition, our results highlight the importance of the morphotypes for the efficient expression and secretion of heterologous recombinant proteins when *P. tricornutum* is intended to be used as a cell biofactory (Butler et al., 2020).

## Conclusion and Future Prospects

To date, our knowledge of the specificity of each morphotype of the model diatom *P. tricornutum* is rather limited. Many questions remain regarding the physiological significance of *P. tricornutum* morphogenesis as well as its mechanism of regulation. Indeed, little attention has been paid to the morphotype in *P. tricornutum* (De Martino et al., 2011; Ovide et al., 2018; Song et al., 2020). The results of the present study demonstrate that the oval morphotype appears to be unique and present a specific metabolic network compared to the fusiform and triradiate morphotypes. Moreover, our results confirm that the oval cells are secreting more proteins in the culture medium as previously suggested by Song et al. (2020) and Galas et al. (2021). This characteristic property in oval cells offers new and highly attractive prospects for the development of biopharmaceuticals in the diatom cell factory. Thus, it can be envisioned that the production yield of biopharmaceuticals like monoclonal antibodies, which is currently limited to a few micrograms per liter in *P. tricornutum* (Hempel et al., 2011; Hempel and Maier, 2012; Vanier et al., 2015, 2018; Hempel et al., 2017) could be increased by expression in *P. tricornutum* oval cells. This could be attempted by transforming oval cells or switching transformed fusiform cells within oval cells as previously reported (Tesson et al., 2009). Taking into account the specificity of the oval morphotype could help in the near future to further optimize the production of biopharmaceuticals in the diatom cell factory.

On the other hand, the presence of molecules with antimicrobial properties (Desbois et al., 2009; Prestegard et al., 2015; Butler et al., 2020) and of proteins involved in the biosynthesis of terpenoids highlight new potentialities of application for *P. tricornutum* such as protector of plant immunity. Cell-specific fractions from *P. tricornutum*, such as fatty acids or volatile organic compounds, were reported to possess antimicrobial activities against bacteria (Desbois et al., 2008, 2009; Prestegard et al., 2015). In addition, *P. tricornutum* is able to synthesized α(1-8)-cineole, α-pinene, limonene and also β(1-3)-glucanases that are present in higher concentrations in the oval morphotype (Prestegard et al., 2015; Buhmann et al., 2016). Such molecules are reported to be involved in plant defense response against plant pathogens (Balasubramanian et al., 2012; Lackus et al., 2018). In this context, the presence of a higher number of specific proteins suggests the potential of the oval cells to be good candidates for finding new elicitors.

## Data Availability Statement

The original contributions presented in the study are publicly available. This data can be found here: The analyzed data are presented within the different tables and figures of this manuscript. Raw proteomics data are available in the MassIVE database, under accession number MSV000086835 (ftp://massive.ucsd.edu/MSV000086835/).

## Author Contributions

MB planned and designed the research. CC, BG, PC, CB, and JH performed the experiments. CC, BG, M-LW-B, FT, PC, and MB collected and analyzed the data. MB and CC interpreted the data. MB, CC, and PC wrote the manuscript. All authors have read and agreed on the manuscript prior to the submission.

## Conflict of Interest

The authors declare that the research was conducted in the absence of any commercial or financial relationships that could be construed as a potential conflict of interest.

## Publisher’s Note

All claims expressed in this article are solely those of the authors and do not necessarily represent those of their affiliated organizations, or those of the publisher, the editors and the reviewers. Any product that may be evaluated in this article, or claim that may be made by its manufacturer, is not guaranteed or endorsed by the publisher.

## Footnotes

1 https://www.matrixscience.com/, Boston, United States.

2
https://www.uniprot.org/proteomes/UP000000759

3 Waters corporation, https://www.nonlinear.com/

4
https://www.Blast2GO.com

5
http://protists.ensembl.org/Phaeodactylum_tricornutum/Info/Index

6
https://www.ebi.ac.uk/interpro/search/sequence/

7
http://www.pantherdb.org

8
https://services.healthtech.dtu.dk/service.php?SignalP-5.0

9
https://services.healthtech.dtu.dk/service.php?NetNGlyc-1.0
