# Supplementary material for: Comparative Proteomic Analysis of the Diatom Phaeodactylum tricornutum Reveals New Insights Into Intra- and Extra-Cellular Protein Contents of Its Oval, Fusiform, and Triradiate Morphotypes
Source: Front Plant Sci. 2022 Mar 21;13:673113. doi: 10.3389/fpls.2022.673113 (PMC8977783; doi:10.3389/fpls.2022.673113)
Supplement: Supplementary file 1 [file Data_Sheet_1.docx]

Supplementary Material

# Supplementary Data

Supplementary Material has been submitted as excel files in addition to the supplementary Figures and Tables listed hereafter. This include the following files:

- Supplementary data 1: Output from Blast2go analysis for the fusiform morphotype
- Supplementary data 2: Output from Blast2go analysis for the triradiate morphotype
- Supplementary data 3: Output from the iTRAQ® analysis
- Supplementary data 4: Details of the secreted proteins identified as unique to either the oval, fusiform and triradiate morphotypes, respectively. This dataset is linked to the Venn diagram presented in Figure.
- Supplementary data 5: Prediction of the signal peptide and N-glycosylation consensus site present on the secreted proteins identified in the different morphotypes. Relationship between these data and the information retrieve from Dorrell et al., 2021 regarding horizontal gene transfer in *P. tricornutum*.
- Supplementary data 6: comparison of the proteomic dataset from this study to the transcriptomic data published previously in Ovide et al., 2018.

# Supplementary Figures and Tables

## Supplementary Figures


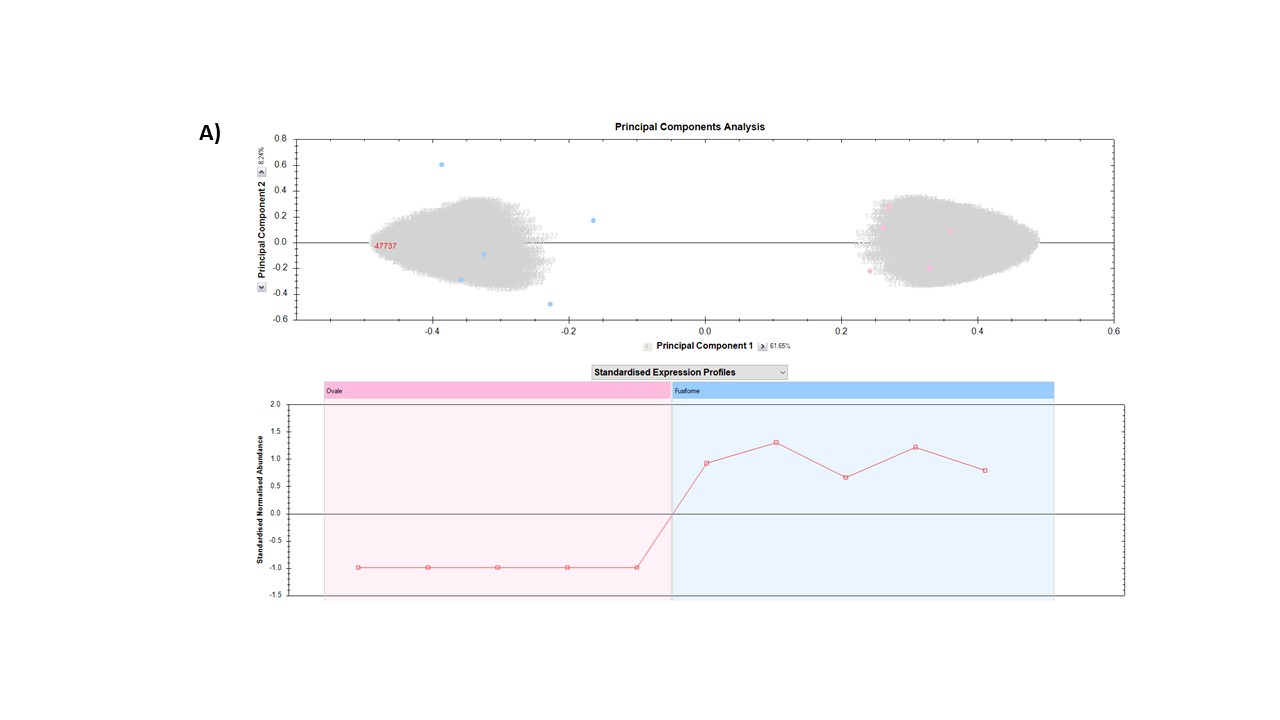

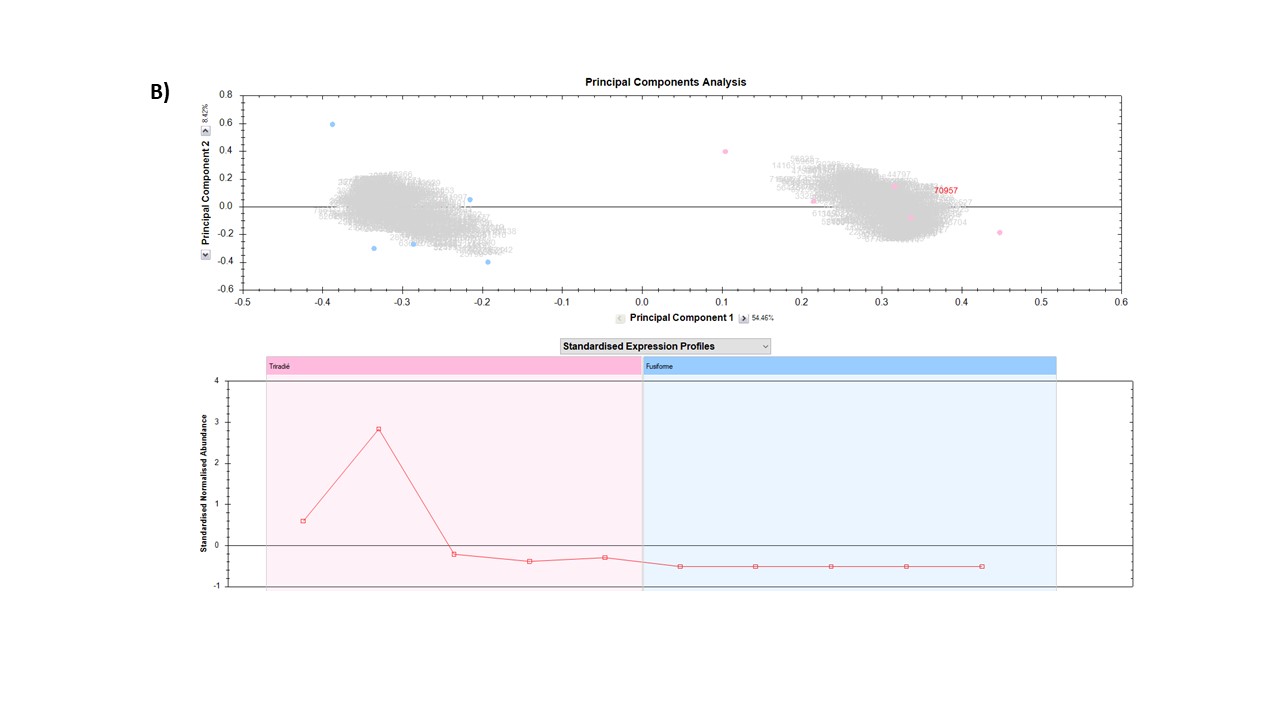


**Supplementary Figure S1:** Principal component analysis (PCA) of the differential expression of proteins between (A) ovale versus fusiform and (B) triradiate versus fusiform. The fusiform morphotype was used as a reference as previously described (De Martino et al., 2007; De Martino et al. 2011; Ovide et al., 2018).

**
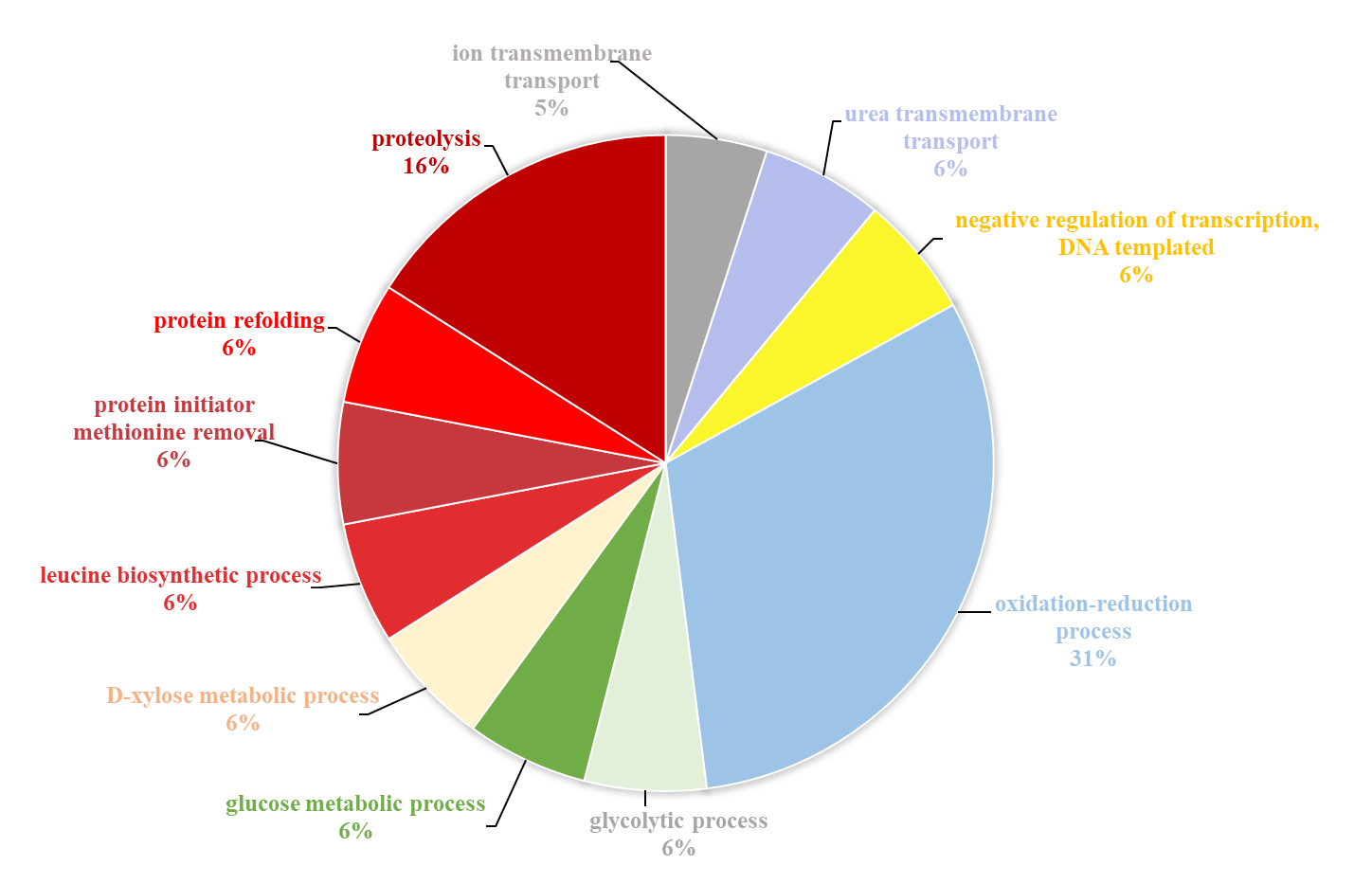
**

**Supplementary Figure S2:** Pie chart representing the biological processes in which the 32 proteins unique to the fusiform morphotype are involved in.


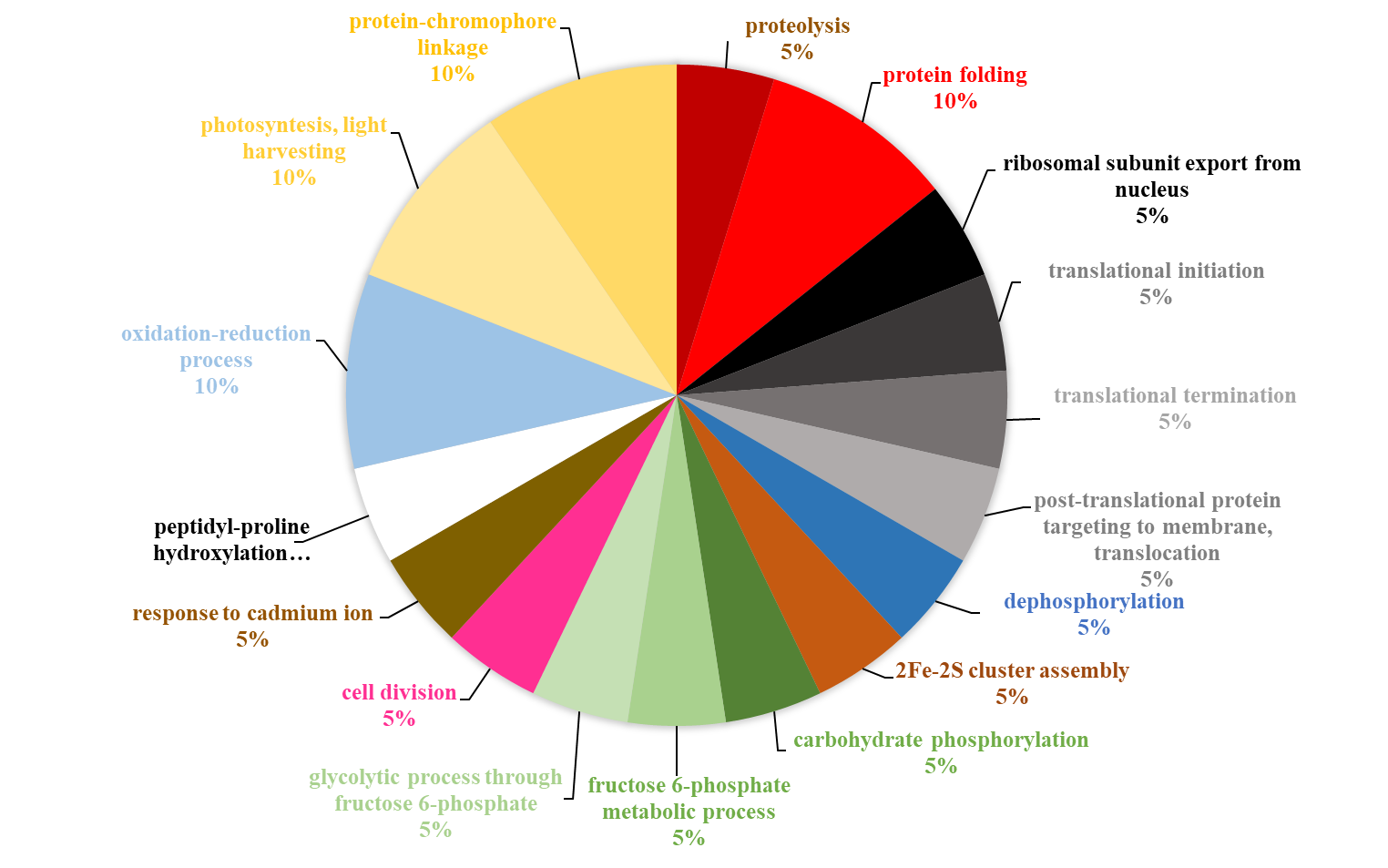


**Supplementary Figure S3:** Pie chart representing the biological processes in which the 16 proteins unique to the triradiate morphotype are involved in.

## Supplementary Tables

**Supplementary table I:** List of the 16 proteins unique to the triradiate morphotype.


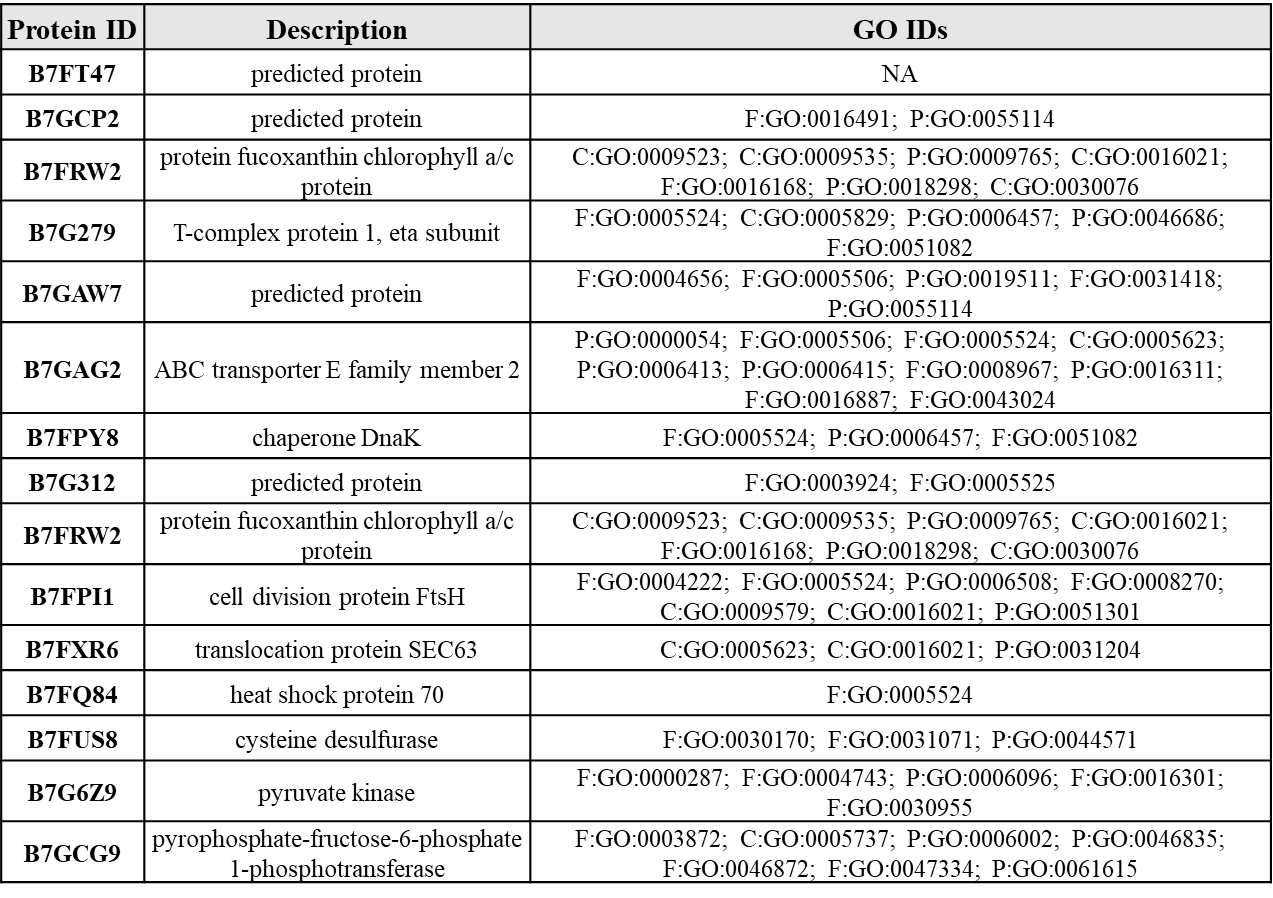


**Supplementary table II:** Proteins that are differentially expressed when comparing the iTRAQ® and label free results


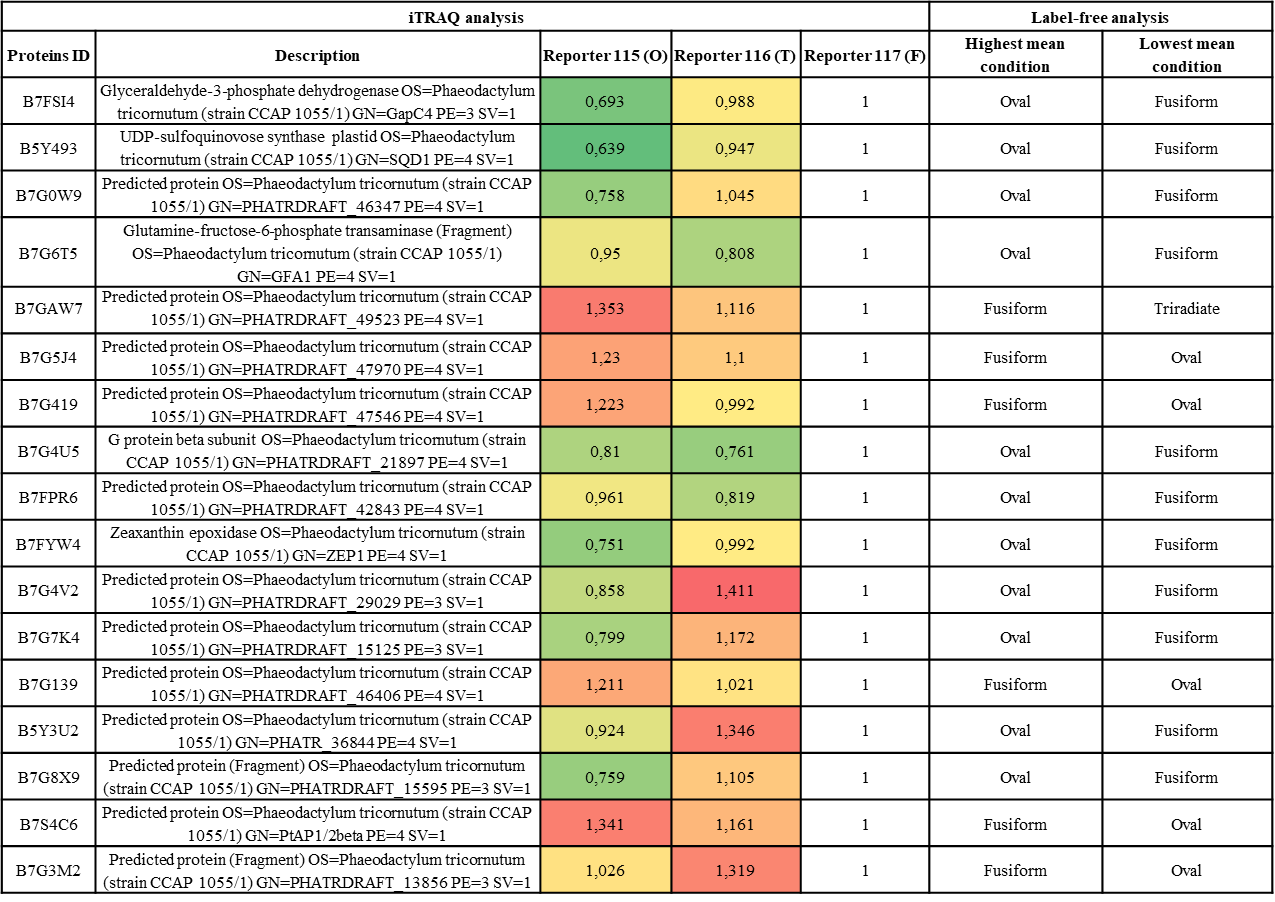


**
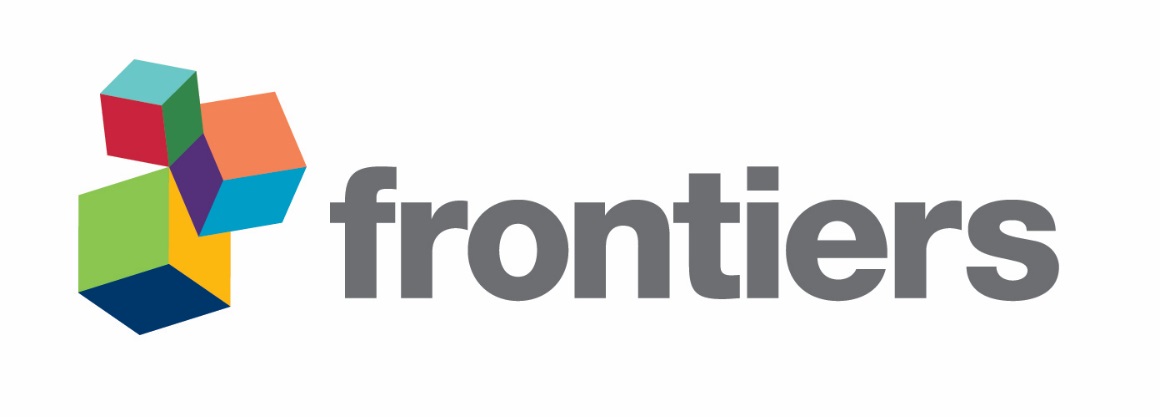
**
